# Crystal structure of the 1:1 adduct of 2,3-diphenyl-3,4,5,6-tetra­hydro-2*H*-1,3-thia­zin-4-one with tri­phenyl­tin chloride

**DOI:** 10.1107/S2056989016001730

**Published:** 2016-02-03

**Authors:** Hemant P. Yennawar, Ryan Fox, Lee J. Silverberg

**Affiliations:** a Department of Chemistry, Pennsylvania State University, University Park, PA 16802, USA; b Pennsylvania State University, Schuylkill Campus, 200 University Drive, Schuylkill Haven, PA 17972, USA

**Keywords:** crystal structure, thia­zine, thia­zinone, adduct structure, envelope pucker, trigonal–bipyramidal, tin

## Abstract

The title adduct, chlorido­(2,3-diphenyl-3,4,5,6-tetra­hydro-2*H*-1,3-thia­zin-4-one-κ*O*)tri­phenyl­tin, [Sn(C_6_H_5_)_3_Cl(C_16_H_15_NOS)], resulted from reaction of 2,3-diphenyl-3,4,5,6-tetra­hydro-2*H*-1,3-thia­zin-4-one with tri­phenyl­tin chloride. The thia­zine ring has an envelope conformation with the S atom forming the flap. The mol­ecule has five phenyl rings, two of them attached to the thia­zine ring at positions 2 and 3, and three in coordination with the Sn^IV^ atom. The three rings of the tri­phenyl­tin group are involved in intra­molecular inter­actions of different types, C—H⋯O, edge-to-face (or T-type) π–π inter­actions with the 3-phenyl ring of the thia­zine, T-type inter­actions with both phenyl rings of the thia­zine *etc*. On the other hand, all the phenyl rings participate in inter­molecular π–π inter­actions. There is one instance of a ‘parallel-displaced’-type inter­action extending continuously along the *a*-axis direction and seven instances of T-type inter­actions stabilizing the crystal lattice.

## Abstract 2

In the adduct resulting from the reaction of 2,3-diphenyl-3,4,5,6-tetra­hydro-2*H*-1,3-thia­zin-4-one with tri­phenyl­tin chloride, the three rings of the tri­phenyl­tin group are involved in intra­molecular inter­actions of different types, and all the phenyl rings participate in inter­molecular π–π inter­actions.

## Chemical context   

Eng and coworkers have reported the synthesis and fungicidal activity of 1:1 complexes of tri­phenyl­tin chloride complexes with five-membered 1,3-thia­zolidin-4-ones (Smith *et al.*, 1995 ▸; Eng *et al.*, 1996 ▸, 1998 ▸), including a crystal structure of 2,3-diphenyl-1,3-thia­zolidin-4-one (**1**) (Scheme 1) (Smith *et al.*, 1995 ▸). Tahara *et al.* have reported the preparation of similar 1:1 adducts of tri­phenyl­tin chloride with lactams, including the six-membered valerolactam (**2**) (Scheme 1) (Tahara *et al.*, 1987 ▸). They did not report a crystal structure of (**2**), but did report a crystal structure of the adduct of the seven-membered caprolactam. All of the complexes reported by Tahara and Eng bind through the carbonyl oxygen atom to the central tin atom and adopt a distorted trigonal–bipyramidal geometry around the tin atom, with the heterocycle and chlorine in axial positions.
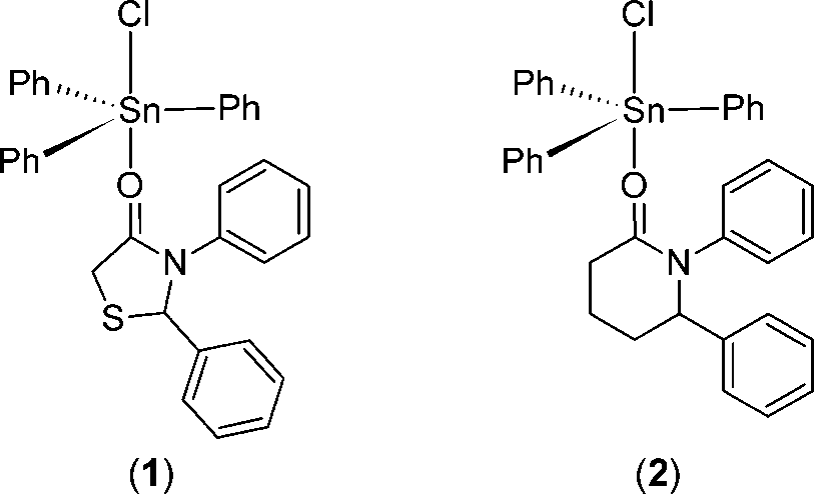


We have recently reported a variety of six- and seven-membered 2,3-diaryl-1,3-thi­aza-4-one heterocycles, including 2,3-diphenyl-3,4,5,6-tetra­hydro-2*H*-1,3-thia­zin-4-one (**3**) (Yennawar & Silverberg, 2014 ▸; Silverberg, *et al.*, 2015 ▸). Herein, we report the synthesis and crystal structure of the 1:1 adduct (**4**) resulting from reaction of (**3**) with tri­phenyl­tin chloride (Scheme 2), which to the best of our knowledge is the first preparation of a tin complex of any 2,3-disubstituted-1,3-thia­zin-4-one heterocycle [Eng *et al.* (1996 ▸) reported the adduct of 3-phenyl-1,3-thia­zinane-2,4-dione]. Crystals for X-ray crystallographic analysis were grown by slow evaporation of the adduct solution in cyclo­hexane. 
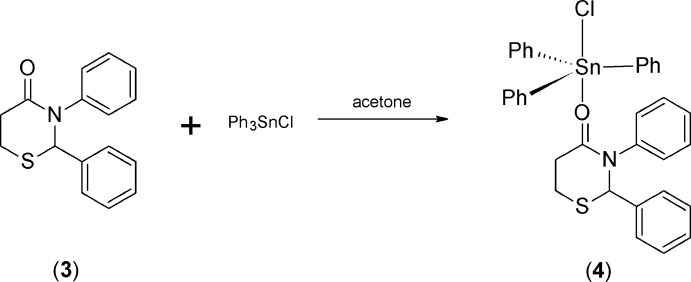


## Structural commentary   

**Figure 1:**
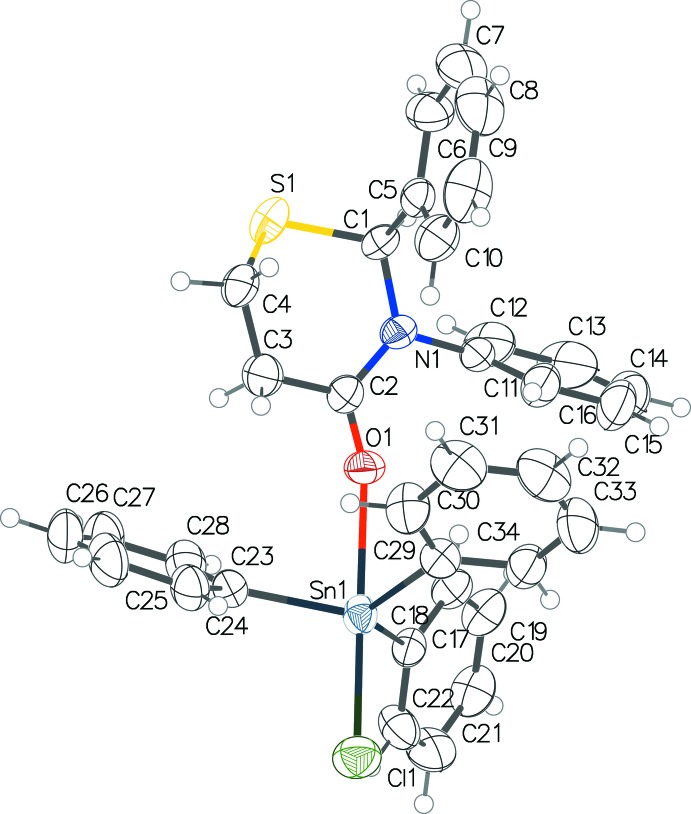
Ellipsoid plot (50% probability level for non-H atoms) of the title compound (**4**).

The molecular structure obtained (Fig. 1 ▸) is similar to that reported for (**1**) (Smith *et al.*, 1995 ▸). It is a 1:1 complex, with the carbonyl oxygen in (**3**) bound to the tin atom. The tin atom is penta­coordinate with a distorted trigonal–bipyramidal geometry (Table 1 ▸), the apical axis being the O–Sn–Cl line. Chlorine and (**3**) are in the axial positions and the three phenyl groups are equatorial. The C—Sn, Cl—Sn, and C—O bond lengths are similar to those in (**1**).

The current crystal structure (**4**) exhibits an envelope conformation for the thia­zine ring with the sulfur atom forming the flap, similar to (**3**) (Yennawar & Silverberg, 2014 ▸, 2015 ▸). The structure has a C—H⋯O type inter­action between the only oxygen atom (O1) and a phenyl carbon C18 of the same mol­ecule. Extensive intra- and inter­molecular ring inter­actions influence the structure of the mol­ecule as well as the crystal packing. Both parallel-displaced and T-shaped inter­actions, analyzed using *PLATON* (Spek, 2009 ▸) have been observed and are discussed below in Section 3.

## Supra­molecular Features   

**Figure 2:**
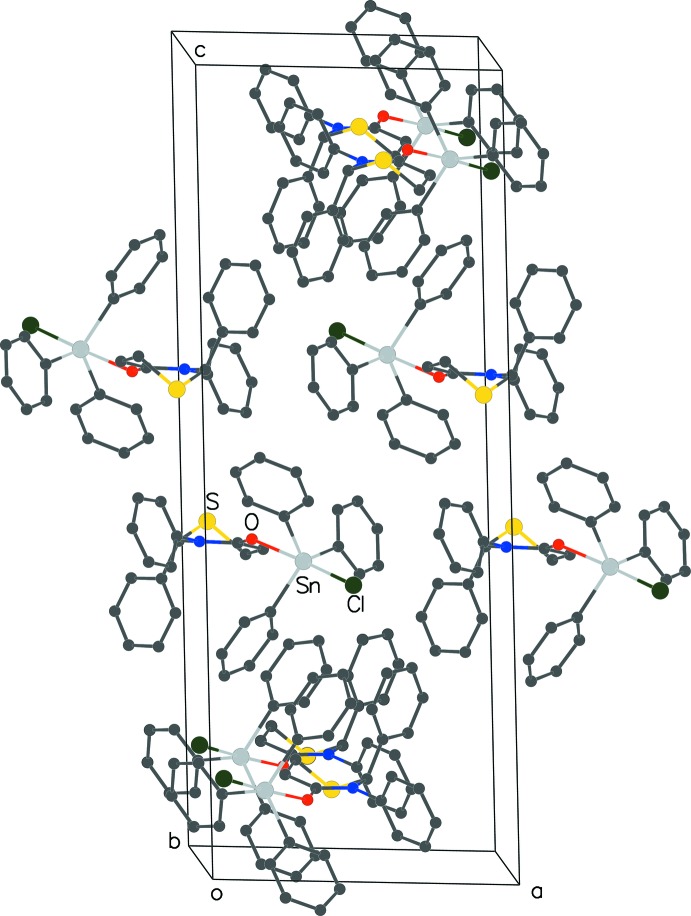
The packing of the title compound (**4**).

The adduct has a thia­zine ring (ring-1) and five phenyl rings (rings-2 and ring-3 attached at positions 2 and 3 of the thia­zine and rings 4, 5 and 6 of the tri­phenyl­tin moiety). The intra­molecular inter­actions between all six rings influence orientation of the phenyl rings and the inter­molecular inter­actions of the five phenyl rings stabilize the crystal lattice (Fig. 2 ▸).

*Intra­molecular inter­actions –* Carbon C18 of ring-4 has a C—H⋯O type inter­action with the only oxygen O1 in the mol­ecule [C18⋯O1 = 3.017 (4) Å; C18—H18⋯O1 = 124°]. The same carbon C18 is at a distance of 3.8287 (7) Å from the centroid of ring-3, resulting in a T-type π–π ring-4 ⋯ ring-3 inter­action. Ring-6 has T-type inter­actions with both (ring-2 and ring-3) phenyl rings of the thia­zine with inter-centroid distances of 5.112 (1) with ring-3 and 5.954 (1) Å with ring-2. The C3 atom of the thia­zine ring is 3.5235 (6) Å from the centroid of ring-5, resulting in a C—H⋯π inter­action. Thus all six rings, aromatic and non-aromatic, participate in influencing the structure of the mol­ecule.

*Inter­molecular inter­actions –* The five phenyl rings inter­act extensively with the phenyl rings of the neighboring mol­ecules in the lattice. Of the eight such π–π inter­actions, one belongs to the parallel-displaced type and seven are of the T-type. In the parallel-displaced inter­action, ring-3 and ring-5 of a mol­ecule inter­act respectively with ring-5 and ring-3 of mol­ecules on opposite sides, forming a continuous chain along the *a-*axis direction. The distance between the centroids of these partially overlapping rings is 3.8627 (7) Å and the dihedral angle is 2° between the ring planes. Seven T-type inter­actions stabilize the lattice further with centroid distances ranging from 5.1688 (9) to 5.8599 (10) Å and the dihedral angles of 69° to 89°. Rings 2, 5 and 6 participate in three inter­actions each, ring-4 in two and ring-3 in one. The intra- and inter­molecular π–π inter­actions are listed in Table 2 ▸ **.**

## Database Survey   

The crystal structure of tri­phenyl­tin chloride has also been reported (Tse *et al.*, 1986 ▸; Bokii *et al.*, 1970 ▸).

## Synthesis and crystallization   

Adduct (**4**) was prepared by reacting an equivalent each of tri­phenyl­tin chloride (Ph_3_SnCl) and 2,3-diphenyl-3,4,5,6-tetra­hydro-2*H*-1,3-thia­zin-4-one (**3**) (Yennawar & Silverberg, 2014 ▸) in acetone (Scheme 2) (Smith *et al.*, 1995 ▸; Cannon, 2015 ▸). The solvent was removed and the solid was recrystallized from ligroin.

**General:** Tri­phenyl­tin chloride was purchased from Sigma–Aldrich (St. Louis, MO). Ligroin (363–383 K b.p. range) was purchased from Fisher Chemical (Pittsburgh, PA). Low-water acetone was purchased from J. T. Baker (Center Valley, PA). Melting points were determined with a Thomas Hoover Capillary Melting Point Apparatus (Arthur H. Thomas Co., Philadelphia, PA).

*1:1 Adduct* (***4**) of 2,3-Diphenyl-3,4,5,6-tetra­hydro-2H-1,3-thia­zin-4-one* (***3**) with tri­phenyl­tin chloride:* A two-neck 10 mL round-bottom flask and a 5 mL round-bottom flask with stir bars were oven-dried, fitted with septa, and cooled under N_2_. Tri­phenyl­tin chloride (0.1427 g, 0.37 mmol) was added to the 10 mL flask. 2,3-Diphenyl-3,4,5,6-tetra­hydro-2*H*-1,3-thia­zin-4-one **3** (0.100 g, 0.37 mmol) was added to the 5 mL flask. 2.5 mL of low-water acetone was added to each flask and each solution was stirred. The contents of the 5 mL flask were transferred to the 10 mL flask dropwise by syringe over a period of 30 minutes. After two h of stirring, the stirrer was turned off. The solution was slightly hazy. After four days, the solution was transferred to a 50 mL round-bottom flask with acetone and concentrated under vacuum to a white solid. Recrystallization from ligroin produced (**4)** as a white powder (0.1086 g, 45%), m.p. 405–407 K. Crystals for X-ray crystallography were grown by slow evaporation from cyclo­hexane.

## Refinement   

Crystal data, data collection and structure refinement details are summarized in Table 3 ▸. Hydrogen atoms were placed geometrically to ride on the carbon atoms during refinement with C—H distances of 0.97 Å (>CH_2_) and 0.93 Å (–CH_arom_) and with *U*_iso_(H) = 1.2*U*_eq_(C).

## Supplementary Material

Crystal structure: contains datablock(s) I. DOI: 10.1107/S2056989016001730/bg2579sup1.cif

Structure factors: contains datablock(s) I. DOI: 10.1107/S2056989016001730/bg2579Isup2.hkl

Supporting information file. DOI: 10.1107/S2056989016001730/bg2579Isup4.mol

CCDC reference: 1450325

Additional supporting information:  crystallographic information; 3D view; checkCIF report

## Figures and Tables

**Table 1:** Selected geometric parameters (Å, °)

C17—Sn1	2.140 (3)	Cl1—Sn1	2.4558 (10)
C23—Sn1	2.123 (3)	O1—Sn1	2.512 (2)
C29—Sn1	2.134 (4)		
			
C23—Sn1—C17	117.50 (12)	C29—Sn1—C17	119.48 (12)
C23—Sn1—C29	117.48 (12)	Cl1—Sn1—O1	178.00 (6)

**Table 2:** Intra- and inter­molecular π–π inter­actions (Å, °) *Cg*2, *Cg*3, *Cg*4, *Cg*5 and *Cg*6 are the centroids of the C5–C10, C11–C16, C17–C22, C23–C28, and C29–C34 rings, respectively.

*CgI*⋯*CgJ*	*Cg*⋯*Cg*	Dihedral angle	Comment
*Cg*3⋯*Cg*4	5.1455 (9)	85	Intra – T-type
*Cg*6⋯*Cg*2	5.9538 (10)	83	Intra – T-type
*Cg*6⋯*Cg*3	5.1126 (9)	50	Intra – T-type
*Cg*2⋯*Cg*5^i^	5.3346 (9)	84	Inter – T-type
*Cg*2⋯*Cg*2^ii^	5.8549 (10)	89	Inter – T-type
*Cg*2⋯*Cg*6^iii^	5.5685 (10)	83	Inter – T-type
*Cg*3⋯*Cg*5^i^	3.8627 (7)	2	Inter – parallel-displaced
*Cg*3⋯*Cg*4^iv^	5.7753 (10)	85	Inter – T-type
*Cg*4⋯*Cg*5^v^	5.1688 (9)	86	Inter – T-type
*Cg*5⋯*Cg*6^vi^	5.8599 (10)	89	Inter – T-type
*Cg*6⋯*Cg*6^vii^	5.5050 (10)	69	Inter – T-type

Symmetry codes: (i) 1 + *x*, *y*, *z*; (ii) 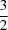 − *x*, −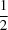 + *y*, 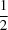 − *z*; (iii) *x*, 1 + *y*, *z*; (iv) 1 − *x*, 2 − *y*, −*z*; (v) −*x*, 2 − *y*, −*z*; (vi) 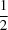 − *x*, 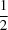 + *y*, 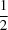 − *z*; (vii) 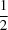 − *x*, −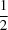 + *y*, 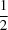 − *z*.

**Table 3:** Experimental details

Crystal data	
Chemical formula	[Sn(C_6_H_5_)_3_Cl(C_16_H_15_NOS)]
*M*_r_	654.79
Crystal system, space group	Monoclinic, *P*2_1_/*n*
Temperature (K)	298
*a*, *b*, *c* (Å)	10.8454 (19), 9.5675 (16), 28.891 (5)
β (°)	92.886 (3)
*V* (Å^3^)	2994.0 (9)
*Z*	4
Radiation type	Mo *K*α
μ (mm^−1^)	1.04
Crystal size (mm)	0.21 × 0.18 × 0.17

Data collection	
Diffractometer	Bruker SMART APEX CCD area detector
Absorption correction	Multi-scan (*SADABS*; Bruker, 2001 ▸)
*T*_min_, *T*_max_	0.820, 1.0
No. of measured, independent and observed [*I* > 2σ(*I*)] reflections	27794, 7403, 6561
*R*_int_	0.024
(sin θ/λ)_max_ (Å^−1^)	0.667

Refinement	
*R*[*F* ^2^ > 2σ(*F* ^2^)], *wR*(*F* ^2^), *S*	0.048, 0.144, 1.08
No. of reflections	7403
No. of parameters	352
H-atom treatment	H-atom parameters constrained
Δρ_max_, Δρ_min_ (e Å^−3^)	1.14, −1.07

Computer programs: *SMART* and *SAINT* (Bruker, 2001 ▸), *olex2.solve* (Bourhis *et al.*, 2015 ▸), *SHELXL2014* (Sheldrick, 2015 ▸) and *OLEX2* (Dolomanov *et al.*, 2009 ▸).

## supplementary crystallographic information

### Crystal data

**Table:**

[Sn(C_6_H_5_)_3_Cl(C_16_H_15_NOS)]	*F*(000) = 1328
*M**_r_* = 654.79	*D*_x_ = 1.453 Mg m^−^^3^
Monoclinic, *P*2_1_/*n*	Mo *K*α radiation, λ = 0.71073 Å
*a* = 10.8454 (19) Å	Cell parameters from 5487 reflections
*b* = 9.5675 (16) Å	θ = 2.2–28.2°
*c* = 28.891 (5) Å	µ = 1.04 mm^−^^1^
β = 92.886 (3)°	*T* = 298 K
*V* = 2994.0 (9) Å^3^	Block, clear colourless
*Z* = 4	0.21 × 0.18 × 0.17 mm

### Data collection

**Table:**

Bruker SMART APEX CCD area-detector diffractometer	6561 reflections with *I* > 2σ(*I*)
Parallel, graphite monochromator	*R*_int_ = 0.024
phi and ω scans	θ_max_ = 28.3°, θ_min_ = 2.0°
Absorption correction: multi-scan (*SADABS*; Bruker, 2001)	*h* = −14→14
*T*_min_ = 0.820, *T*_max_ = 1.0	*k* = −11→12
27794 measured reflections	*l* = −38→38
7403 independent reflections	

### Refinement

**Table:**

Refinement on *F*^2^	0 restraints
Least-squares matrix: full	Hydrogen site location: inferred from neighbouring sites
*R*[*F*^2^ > 2σ(*F*^2^)] = 0.048	H-atom parameters constrained
*wR*(*F*^2^) = 0.144	*w* = 1/[σ^2^(*F*_o_^2^) + (0.1*P*)^2^] where *P* = (*F*_o_^2^ + 2*F*_c_^2^)/3
*S* = 1.08	(Δ/σ)_max_ = 0.001
7403 reflections	Δρ_max_ = 1.14 e Å^−^^3^
352 parameters	Δρ_min_ = −1.07 e Å^−^^3^

### Special details

**Table:**

Experimental. The data collection nominally covered a full sphere of reciprocal space by a combination of 4 sets of ω scans each set at different φ and/or 2θ angles and each scan (5 s exposure) covering −0.300° degrees in ω. The crystal to detector distance was 5.82 cm.
Geometry. All e.s.d.'s (except the e.s.d. in the dihedral angle between two l.s. planes) are estimated using the full covariance matrix. The cell e.s.d.'s are taken into account individually in the estimation of e.s.d.'s in distances, angles and torsion angles; correlations between e.s.d.'s in cell parameters are only used when they are defined by crystal symmetry. An approximate (isotropic) treatment of cell e.s.d.'s is used for estimating e.s.d.'s involving l.s. planes.
Refinement. None

### Fractional atomic coordinates and isotropic or equivalent isotropic displacement parameters (Å^2^)

**Table:**

	*x*	*y*	*z*	*U*_iso_*/*U*_eq_	
C1	−0.0293 (3)	0.7598 (3)	0.38394 (10)	0.0346 (6)	
H1	−0.0976	0.7618	0.4049	0.042*	
C2	0.1500 (3)	0.5935 (3)	0.38953 (11)	0.0405 (7)	
C3	0.2413 (3)	0.7053 (4)	0.37798 (19)	0.0634 (12)	
H3A	0.2994	0.7149	0.4044	0.076*	
H3B	0.2874	0.6714	0.3524	0.076*	
C4	0.1947 (3)	0.8490 (4)	0.36539 (14)	0.0487 (8)	
H4A	0.1623	0.8498	0.3335	0.058*	
H4B	0.2621	0.9156	0.3683	0.058*	
C5	−0.0856 (3)	0.7891 (3)	0.33611 (12)	0.0390 (7)	
C6	−0.1767 (3)	0.8910 (4)	0.33192 (15)	0.0560 (9)	
H6	−0.2019	0.9362	0.3583	0.067*	
C7	−0.2300 (4)	0.9260 (5)	0.28952 (18)	0.0762 (13)	
H7	−0.2923	0.9929	0.2874	0.091*	
C8	−0.1919 (5)	0.8631 (6)	0.25064 (19)	0.0848 (16)	
H8	−0.2270	0.8884	0.2218	0.102*	
C9	−0.1022 (5)	0.7628 (6)	0.25366 (15)	0.0750 (13)	
H9	−0.0768	0.7202	0.2268	0.090*	
C10	−0.0479 (4)	0.7232 (4)	0.29686 (14)	0.0543 (9)	
H10	0.0123	0.6539	0.2989	0.065*	
C11	−0.0549 (3)	0.5089 (3)	0.39995 (13)	0.0423 (7)	
C12	−0.1050 (4)	0.5019 (4)	0.44224 (15)	0.0628 (10)	
H12	−0.0850	0.5679	0.4650	0.075*	
C13	−0.1872 (4)	0.3932 (6)	0.4506 (2)	0.095 (2)	
H13	−0.2227	0.3869	0.4791	0.114*	
C14	−0.2153 (5)	0.2971 (6)	0.4172 (3)	0.096 (2)	
H14	−0.2706	0.2257	0.4230	0.115*	
C15	−0.1640 (5)	0.3038 (5)	0.3759 (2)	0.087 (2)	
H15	−0.1831	0.2360	0.3536	0.105*	
C16	−0.0835 (4)	0.4096 (4)	0.36625 (16)	0.0620 (10)	
H16	−0.0487	0.4144	0.3375	0.074*	
C17	0.2724 (3)	0.1831 (3)	0.44010 (10)	0.0366 (6)	
C18	0.1561 (3)	0.2141 (3)	0.45627 (12)	0.0430 (7)	
H18	0.1068	0.2818	0.4415	0.052*	
C19	0.1141 (4)	0.1431 (4)	0.49471 (12)	0.0505 (8)	
H19	0.0362	0.1626	0.5051	0.061*	
C20	0.1861 (4)	0.0461 (4)	0.51693 (12)	0.0556 (9)	
H20	0.1580	0.0006	0.5428	0.067*	
C21	0.3013 (4)	0.0143 (5)	0.50130 (14)	0.0627 (10)	
H21	0.3502	−0.0532	0.5164	0.075*	
C22	0.3434 (3)	0.0834 (4)	0.46308 (12)	0.0521 (9)	
H22	0.4210	0.0621	0.4528	0.062*	
C23	0.4673 (3)	0.4534 (3)	0.39319 (12)	0.0410 (7)	
C24	0.5381 (3)	0.5026 (4)	0.35781 (14)	0.0521 (8)	
H24	0.5375	0.4546	0.3298	0.063*	
C25	0.6092 (4)	0.6217 (5)	0.36365 (17)	0.0679 (12)	
H25	0.6536	0.6560	0.3394	0.082*	
C26	0.6136 (4)	0.6895 (5)	0.4061 (2)	0.0752 (14)	
H26	0.6620	0.7691	0.4104	0.090*	
C27	0.5473 (4)	0.6402 (5)	0.44153 (17)	0.0718 (12)	
H27	0.5520	0.6856	0.4700	0.086*	
C28	0.4730 (3)	0.5232 (4)	0.43537 (13)	0.0540 (9)	
H28	0.4269	0.4913	0.4595	0.065*	
C29	0.2290 (3)	0.2921 (3)	0.31778 (12)	0.0425 (7)	
C30	0.2426 (4)	0.3976 (4)	0.28580 (12)	0.0564 (9)	
H30	0.3056	0.4626	0.2909	0.068*	
C31	0.1660 (4)	0.4095 (5)	0.24657 (14)	0.0675 (11)	
H31	0.1755	0.4835	0.2262	0.081*	
C32	0.0757 (4)	0.3115 (6)	0.23774 (15)	0.0703 (12)	
H32	0.0247	0.3175	0.2110	0.084*	
C33	0.0607 (4)	0.2040 (5)	0.26873 (19)	0.0747 (14)	
H33	−0.0004	0.1373	0.2628	0.090*	
C34	0.1362 (4)	0.1950 (4)	0.30866 (15)	0.0548 (9)	
H34	0.1246	0.1230	0.3296	0.066*	
Cl1	0.48733 (9)	0.10626 (10)	0.36005 (4)	0.0572 (2)	
N1	0.0277 (2)	0.6213 (3)	0.38945 (9)	0.0364 (5)	
O1	0.18753 (19)	0.4749 (2)	0.39935 (9)	0.0467 (5)	
S1	0.07553 (9)	0.89817 (9)	0.40313 (3)	0.0494 (2)	
Sn1	0.33979 (2)	0.28758 (2)	0.38094 (2)	0.03769 (10)	

### Atomic displacement parameters (Å^2^)

**Table:**

	*U*^11^	*U*^22^	*U*^33^	*U*^12^	*U*^13^	*U*^23^
C1	0.0426 (15)	0.0265 (13)	0.0354 (14)	−0.0004 (11)	0.0088 (12)	0.0011 (12)
C2	0.0404 (15)	0.0348 (16)	0.0460 (17)	−0.0032 (12)	0.0003 (13)	0.0035 (13)
C3	0.0407 (18)	0.048 (2)	0.102 (3)	−0.0064 (14)	0.010 (2)	0.025 (2)
C4	0.0472 (17)	0.0364 (17)	0.063 (2)	−0.0092 (14)	0.0081 (16)	0.0078 (16)
C5	0.0382 (15)	0.0347 (17)	0.0442 (17)	−0.0067 (11)	0.0012 (13)	0.0046 (12)
C6	0.0513 (19)	0.050 (2)	0.067 (2)	0.0065 (15)	0.0062 (17)	0.0169 (18)
C7	0.061 (2)	0.074 (3)	0.092 (4)	0.000 (2)	−0.016 (2)	0.035 (3)
C8	0.086 (3)	0.094 (4)	0.070 (3)	−0.020 (3)	−0.034 (3)	0.030 (3)
C9	0.100 (4)	0.082 (3)	0.043 (2)	−0.026 (3)	0.000 (2)	−0.004 (2)
C10	0.064 (2)	0.050 (2)	0.049 (2)	−0.0045 (16)	0.0046 (18)	−0.0028 (16)
C11	0.0329 (13)	0.0317 (15)	0.062 (2)	−0.0016 (11)	−0.0009 (13)	0.0102 (14)
C12	0.064 (2)	0.054 (2)	0.073 (3)	0.0030 (18)	0.023 (2)	0.021 (2)
C13	0.068 (3)	0.077 (4)	0.145 (5)	0.010 (3)	0.046 (3)	0.056 (4)
C14	0.056 (3)	0.058 (3)	0.171 (7)	−0.019 (2)	−0.021 (3)	0.057 (4)
C15	0.087 (4)	0.044 (3)	0.126 (6)	−0.023 (2)	−0.044 (4)	0.020 (3)
C16	0.065 (2)	0.0397 (19)	0.079 (3)	−0.0082 (17)	−0.021 (2)	0.0052 (19)
C17	0.0402 (14)	0.0336 (14)	0.0356 (14)	−0.0059 (12)	−0.0020 (12)	0.0004 (12)
C18	0.0505 (18)	0.0370 (18)	0.0420 (17)	−0.0018 (12)	0.0069 (14)	0.0021 (13)
C19	0.064 (2)	0.0436 (19)	0.0446 (17)	−0.0069 (16)	0.0139 (16)	−0.0022 (15)
C20	0.074 (2)	0.054 (2)	0.0391 (17)	−0.0142 (18)	0.0062 (16)	0.0088 (16)
C21	0.061 (2)	0.064 (3)	0.062 (2)	0.0017 (18)	−0.0089 (18)	0.030 (2)
C22	0.0382 (15)	0.063 (2)	0.054 (2)	−0.0041 (15)	−0.0047 (14)	0.0191 (17)
C23	0.0327 (14)	0.0399 (17)	0.0499 (17)	−0.0021 (12)	−0.0033 (12)	0.0022 (14)
C24	0.0392 (16)	0.055 (2)	0.063 (2)	−0.0088 (15)	0.0067 (15)	0.0061 (17)
C25	0.047 (2)	0.074 (3)	0.083 (3)	−0.0201 (19)	0.000 (2)	0.026 (2)
C26	0.057 (2)	0.061 (3)	0.105 (4)	−0.025 (2)	−0.019 (3)	0.008 (3)
C27	0.073 (3)	0.067 (3)	0.073 (3)	−0.013 (2)	−0.019 (2)	−0.012 (2)
C28	0.0516 (19)	0.059 (2)	0.0501 (19)	−0.0090 (16)	−0.0078 (15)	0.0051 (17)
C29	0.0433 (16)	0.0439 (19)	0.0405 (16)	0.0002 (12)	0.0022 (14)	0.0007 (13)
C30	0.062 (2)	0.062 (2)	0.0444 (18)	−0.0131 (17)	0.0004 (16)	0.0128 (17)
C31	0.072 (3)	0.083 (3)	0.048 (2)	0.005 (2)	0.0019 (19)	0.019 (2)
C32	0.070 (3)	0.097 (4)	0.042 (2)	0.011 (2)	−0.0127 (19)	−0.006 (2)
C33	0.062 (3)	0.079 (3)	0.081 (3)	−0.011 (2)	−0.018 (2)	−0.019 (2)
C34	0.059 (2)	0.043 (2)	0.061 (2)	−0.0078 (15)	−0.0093 (18)	0.0049 (16)
Cl1	0.0561 (5)	0.0514 (5)	0.0649 (6)	0.0070 (4)	0.0117 (4)	−0.0012 (4)
N1	0.0383 (12)	0.0287 (13)	0.0422 (13)	0.0004 (10)	0.0021 (10)	0.0055 (10)
O1	0.0378 (11)	0.0341 (12)	0.0684 (15)	0.0050 (9)	0.0044 (10)	0.0107 (11)
S1	0.0655 (5)	0.0362 (4)	0.0466 (4)	−0.0109 (4)	0.0033 (4)	−0.0088 (3)
Sn1	0.03675 (14)	0.03780 (16)	0.03835 (15)	−0.00725 (7)	0.00030 (9)	0.00637 (8)

### Geometric parameters (Å, º)

**Table:**

C1—H1	0.9800	C17—C22	1.375 (5)
C1—C5	1.509 (4)	C17—Sn1	2.140 (3)
C1—N1	1.467 (4)	C18—H18	0.9300
C1—S1	1.814 (3)	C18—C19	1.398 (5)
C2—C3	1.507 (4)	C19—H19	0.9300
C2—N1	1.352 (4)	C19—C20	1.354 (5)
C2—O1	1.234 (4)	C20—H20	0.9300
C3—H3A	0.9700	C20—C21	1.383 (6)
C3—H3B	0.9700	C21—H21	0.9300
C3—C4	1.503 (5)	C21—C22	1.384 (5)
C4—H4A	0.9700	C22—H22	0.9300
C4—H4B	0.9700	C23—C24	1.391 (5)
C4—S1	1.795 (4)	C23—C28	1.388 (5)
C5—C6	1.389 (5)	C23—Sn1	2.123 (3)
C5—C10	1.378 (5)	C24—H24	0.9300
C6—H6	0.9300	C24—C25	1.382 (5)
C6—C7	1.370 (6)	C25—H25	0.9300
C7—H7	0.9300	C25—C26	1.385 (7)
C7—C8	1.357 (8)	C26—H26	0.9300
C8—H8	0.9300	C26—C27	1.365 (7)
C8—C9	1.367 (8)	C27—H27	0.9300
C9—H9	0.9300	C27—C28	1.386 (6)
C9—C10	1.405 (6)	C28—H28	0.9300
C10—H10	0.9300	C29—C30	1.381 (5)
C11—C12	1.364 (5)	C29—C34	1.384 (5)
C11—C16	1.384 (5)	C29—Sn1	2.134 (4)
C11—N1	1.442 (4)	C30—H30	0.9300
C12—H12	0.9300	C30—C31	1.376 (5)
C12—C13	1.398 (6)	C31—H31	0.9300
C13—H13	0.9300	C31—C32	1.370 (6)
C13—C14	1.355 (9)	C32—H32	0.9300
C14—H14	0.9300	C32—C33	1.378 (7)
C14—C15	1.344 (9)	C33—H33	0.9300
C15—H15	0.9300	C33—C34	1.383 (6)
C15—C16	1.375 (6)	C34—H34	0.9300
C16—H16	0.9300	Cl1—Sn1	2.4558 (10)
C17—C18	1.399 (4)	O1—Sn1	2.512 (2)
			
C5—C1—H1	106.2	C18—C19—H19	119.8
C5—C1—S1	111.3 (2)	C20—C19—C18	120.5 (3)
N1—C1—H1	106.2	C20—C19—H19	119.8
N1—C1—C5	114.6 (2)	C19—C20—H20	119.9
N1—C1—S1	111.8 (2)	C19—C20—C21	120.3 (3)
S1—C1—H1	106.2	C21—C20—H20	119.9
N1—C2—C3	121.0 (3)	C20—C21—H21	120.2
O1—C2—C3	119.4 (3)	C20—C21—C22	119.7 (3)
O1—C2—N1	119.6 (3)	C22—C21—H21	120.2
C2—C3—H3A	107.5	C17—C22—C21	121.1 (3)
C2—C3—H3B	107.5	C17—C22—H22	119.4
H3A—C3—H3B	107.0	C21—C22—H22	119.4
C4—C3—C2	119.1 (3)	C24—C23—Sn1	120.6 (3)
C4—C3—H3A	107.5	C28—C23—C24	118.8 (3)
C4—C3—H3B	107.5	C28—C23—Sn1	120.3 (2)
C3—C4—H4A	109.7	C23—C24—H24	119.5
C3—C4—H4B	109.7	C25—C24—C23	121.0 (4)
C3—C4—S1	109.6 (3)	C25—C24—H24	119.5
H4A—C4—H4B	108.2	C24—C25—H25	120.4
S1—C4—H4A	109.7	C24—C25—C26	119.2 (4)
S1—C4—H4B	109.7	C26—C25—H25	120.4
C6—C5—C1	117.6 (3)	C25—C26—H26	119.8
C10—C5—C1	123.0 (3)	C27—C26—C25	120.4 (4)
C10—C5—C6	119.3 (4)	C27—C26—H26	119.8
C5—C6—H6	119.5	C26—C27—H27	119.7
C7—C6—C5	121.0 (4)	C26—C27—C28	120.5 (4)
C7—C6—H6	119.5	C28—C27—H27	119.7
C6—C7—H7	120.0	C23—C28—H28	120.0
C8—C7—C6	120.0 (4)	C27—C28—C23	120.1 (4)
C8—C7—H7	120.0	C27—C28—H28	120.0
C7—C8—H8	119.9	C30—C29—C34	117.6 (3)
C7—C8—C9	120.3 (4)	C30—C29—Sn1	120.8 (3)
C9—C8—H8	119.9	C34—C29—Sn1	121.4 (3)
C8—C9—H9	119.6	C29—C30—H30	119.0
C8—C9—C10	120.8 (5)	C31—C30—C29	122.1 (4)
C10—C9—H9	119.6	C31—C30—H30	119.0
C5—C10—C9	118.6 (4)	C30—C31—H31	120.2
C5—C10—H10	120.7	C32—C31—C30	119.6 (4)
C9—C10—H10	120.7	C32—C31—H31	120.2
C12—C11—C16	120.7 (3)	C31—C32—H32	120.1
C12—C11—N1	120.4 (3)	C31—C32—C33	119.7 (4)
C16—C11—N1	118.9 (3)	C33—C32—H32	120.1
C11—C12—H12	120.7	C32—C33—H33	119.9
C11—C12—C13	118.5 (5)	C32—C33—C34	120.2 (4)
C13—C12—H12	120.7	C34—C33—H33	119.9
C12—C13—H13	119.8	C29—C34—H34	119.6
C14—C13—C12	120.3 (5)	C33—C34—C29	120.7 (4)
C14—C13—H13	119.8	C33—C34—H34	119.6
C13—C14—H14	119.7	C2—N1—C1	125.9 (2)
C15—C14—C13	120.7 (4)	C2—N1—C11	118.2 (2)
C15—C14—H14	119.7	C11—N1—C1	115.7 (2)
C14—C15—H15	119.6	C2—O1—Sn1	145.1 (2)
C14—C15—C16	120.7 (5)	C4—S1—C1	94.74 (15)
C16—C15—H15	119.6	C17—Sn1—Cl1	96.85 (9)
C11—C16—H16	120.5	C17—Sn1—O1	84.81 (10)
C15—C16—C11	119.0 (5)	C23—Sn1—C17	117.50 (12)
C15—C16—H16	120.5	C23—Sn1—C29	117.48 (12)
C18—C17—Sn1	121.2 (2)	C23—Sn1—Cl1	98.15 (9)
C22—C17—C18	118.6 (3)	C23—Sn1—O1	82.03 (10)
C22—C17—Sn1	120.3 (2)	C29—Sn1—C17	119.48 (12)
C17—C18—H18	120.1	C29—Sn1—Cl1	98.63 (9)
C19—C18—C17	119.8 (3)	C29—Sn1—O1	79.56 (10)
C19—C18—H18	120.1	Cl1—Sn1—O1	178.00 (6)
			
C1—C5—C6—C7	178.3 (3)	C23—C24—C25—C26	2.6 (6)
C1—C5—C10—C9	−177.0 (3)	C24—C23—C28—C27	0.5 (5)
C2—C3—C4—S1	41.8 (5)	C24—C25—C26—C27	−0.8 (7)
C3—C2—N1—C1	−7.4 (5)	C25—C26—C27—C28	−1.1 (7)
C3—C2—N1—C11	178.8 (4)	C26—C27—C28—C23	1.2 (6)
C3—C2—O1—Sn1	−38.6 (6)	C28—C23—C24—C25	−2.5 (5)
C3—C4—S1—C1	−63.2 (3)	C29—C30—C31—C32	−2.3 (7)
C5—C1—N1—C2	101.4 (3)	C30—C29—C34—C33	0.3 (6)
C5—C1—N1—C11	−84.7 (3)	C30—C31—C32—C33	1.5 (7)
C5—C1—S1—C4	−73.3 (2)	C31—C32—C33—C34	0.2 (7)
C5—C6—C7—C8	−1.6 (7)	C32—C33—C34—C29	−1.0 (7)
C6—C5—C10—C9	0.7 (5)	C34—C29—C30—C31	1.4 (6)
C6—C7—C8—C9	1.3 (8)	N1—C1—C5—C6	157.5 (3)
C7—C8—C9—C10	−0.1 (8)	N1—C1—C5—C10	−24.8 (4)
C8—C9—C10—C5	−0.9 (7)	N1—C1—S1—C4	56.3 (2)
C10—C5—C6—C7	0.5 (5)	N1—C2—C3—C4	−1.1 (6)
C11—C12—C13—C14	0.4 (7)	N1—C2—O1—Sn1	141.6 (3)
C12—C11—C16—C15	0.4 (5)	N1—C11—C12—C13	178.4 (3)
C12—C11—N1—C1	−70.3 (4)	N1—C11—C16—C15	−178.8 (3)
C12—C11—N1—C2	104.1 (4)	O1—C2—C3—C4	179.1 (4)
C12—C13—C14—C15	0.6 (8)	O1—C2—N1—C1	172.4 (3)
C13—C14—C15—C16	−1.1 (8)	O1—C2—N1—C11	−1.4 (5)
C14—C15—C16—C11	0.6 (7)	S1—C1—C5—C6	−74.4 (3)
C16—C11—C12—C13	−0.9 (6)	S1—C1—C5—C10	103.3 (3)
C16—C11—N1—C1	108.9 (3)	S1—C1—N1—C2	−26.4 (4)
C16—C11—N1—C2	−76.7 (4)	S1—C1—N1—C11	147.5 (2)
C17—C18—C19—C20	1.1 (5)	Sn1—C17—C18—C19	179.3 (3)
C18—C17—C22—C21	0.2 (6)	Sn1—C17—C22—C21	−179.7 (3)
C18—C19—C20—C21	−1.2 (6)	Sn1—C23—C24—C25	171.5 (3)
C19—C20—C21—C22	0.8 (6)	Sn1—C23—C28—C27	−173.4 (3)
C20—C21—C22—C17	−0.3 (6)	Sn1—C29—C30—C31	−174.7 (3)
C22—C17—C18—C19	−0.6 (5)	Sn1—C29—C34—C33	176.4 (3)
